# Outcome of multidisciplinary treatment of peripheral primitive neuroectodermal tumor

**DOI:** 10.1038/s41598-020-72680-6

**Published:** 2020-09-24

**Authors:** Yidi Liu, Yan Yuan, Fuquan Zhang, Ke Hu, Jie Qiu, Xiaorong Hou, Junfang Yan, Xin Lian, Shuai Sun, Zhikai Liu, Jie Shen

**Affiliations:** Department of Radiation Oncology, Peking Union Medical College Hospital. Chinese Academy of Medical Sciences & Peking Union Medical College, No.1 Shuaifuyuan Wangfujing, Dongcheng District, Beijing, 100730 People’s Republic of China

**Keywords:** Sarcoma, Radiotherapy

## Abstract

Peripheral primitive neuroectodermal tumors (PNETs) constitute very rare and aggressive malignancies. To date, there are no standard guidelines for management of peripheral PNETs due to the paucity of cases arising in various body sites. Therapeutic approach is derived from Ewing sarcoma family, which currently remains multimodal. Our study retrospectively analyzed 86 PNET patients from February 1, 1998 to February 1, 2018 at Peking Union Medical College Hospital with an additional 75 patients from review of literature. The clinicopathologic and treatment plans associated with survival was investigated. Surgery, chemotherapy, female sex, small tumor size, no lymph node metastasis, R0 surgical resection, (vincristine + doxorubicin + cyclophosphamide)/(isophosphamide + etoposide) regimen, and more than 10 cycles of chemotherapy were associated with improved overall survival in univariate analysis. Surgery, more than 10 cycles of chemotherapy, and small tumor size were independent prognostic factors for higher overall survival. Our data indicates that multimodal therapy is the mainstay therapeutic approach for peripheral PNET.

## Introduction

Primitive neuroectodermal tumor (PNET) is a rare and highly malignant small round cell tumor and its concept was first introduced by Hart et al*.*in 1973^1^. Annual incidence of this tumor is approximately 0.2–0.4 per 100,000 and it most commonly occurs in children and young adults with a slight male predominance^2–4^. This tumor, which belongs to the Ewing’s sarcoma family, mainly arises from primary neuroepithelia and possesses multidirectional differentiation potential^3^. Because of its embryonic origin, PNET may arise in any organ^5^. Under light microscopy, PNET exhibits diffuse sheets, lobules or focal nests of small round cells with deeply stained round, oval, or irregular nuclei. Increased mitotic figures and neural Homer-Wright rosettes can be observed. Based on the tissue of origin of the tumors, they are divided into peripheral PNET (pPNET) and central PNET (cPNET). pPNET mainly arises in the skeletal system and soft tissues, while the occurrence of cPNET is primarily intracranial and intraspinal. pPNET may also be present in visceral body sites such as heart, lung, genital organs, kidney, pancreas and palate^6,7^. The diagnosis of PNET is based on histological and immunohistochemical examination of tissue sample. PNETs are usually positive for CD99, neuron-specific enolase (NSE), CD56 and negative for markers for epithelia, lymphoid tissue, musculoskeletal tissue and melanoma. At least two of the above three markers should be positive to make a diagnosis of PNET^8^.

Due to the rarity of peripheral PNET, it is unlikely to conduct prospective studies to reveal the impact of clinical and treatment plans on survival prognosis. Furthermore, according to our literature search, most articles on pPNET were case reports or small case-series, making it difficult to draw conclusions on overall management tailored to this disease. Therefore, to elucidate the prognosis of pPNET and improve the therapeutic approaches, the current study collectively investigated 161 PNET patients both from our own institution and from literature review, and analyzed the survival impacts of the clinical and treatment features of these patients.

## Results

### Patient characteristics

**Figure 1:**
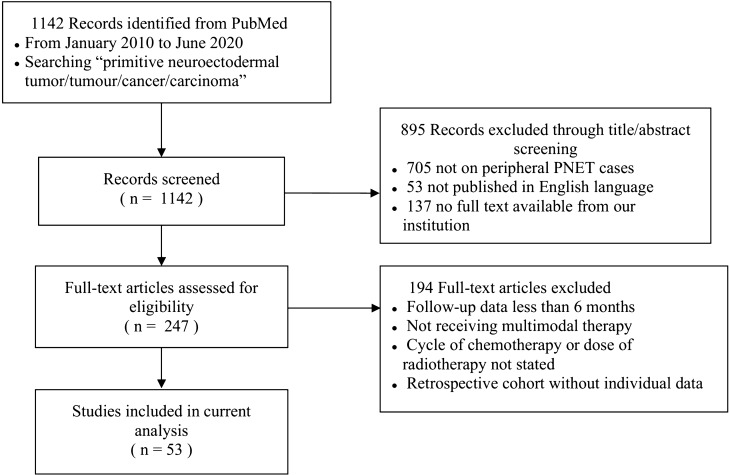
Flowchart of studies included in the analysis. 1142 articles were initially identified from Pubmed database, and 53 were finally included in the current analysis.

Of 161 patients, 89 were female and 72 were male, with a male/female ratio of 0.81:1. The median age of diagnosis was 24 years (range 1–78 years). 38.5% patients were ≤ 18 years at the age of diagnosis, 35.4% were 18–35 years, and 26.1% were > 35 years. In terms of the location of primary tumor occurrence, 40 cases occurred in trunk, extremities and bones, 46 in abdominal and pelvic region (including kidney), 35 in the urogenital system, 16 in thoracic viscera, 20 in head and neck, and 4 in the spine. Based on the location, 90 cases occurred in visceral organs and 71 occurred in non-visceral organs. 86 patients were enrolled from Peking Union Medical College Hospital (PUMCH), and the other 75 cases were extracted from 53 articles in literature (See literature search result in the Supplementary Table S1 and S2). A flowchart of article screening for the current analysis is illustrated in Fig. 1. Features of the studied population are summarized in Table 1. Comparison between our patients and previously reported cases is summarized in Table 2.

**Table 1:** Clinical and treatment features with associated OS.

Variables	N	Univariate		Multivariate	
		Median OS (month)	*P* value	Hazard ratio	*P* value
**Age at diagnosis**					
> 35 years	42	12	0.449		
18–35 years	57	17			
≤ 18 years	62	18			
**Sex**					
Female	89	21	0.009	0.581	0.088
Male	72	13			
**Tumor site**					
Visceral	90	15	0.138		
Non-visceral	71	18			
**Surgery**					
Yes	136	18	< 0.001	0.362	0.005
No	25	10			
**Chemotherapy**					
Yes	135	17	0.001	2.547	0.225
No	26	13.5			
**Radiotherapy**					
Yes	67	19	0.235		
No	94	14			
**Tumor diameter**					
≤ 5 cm	34	17	0.049	1.393	0.029
> 5 cm, ≤ 10 cm	58	22.5			
> 10 cm	45	14			
Unknown	24	14.5			
**Lymph node status**					
No metastasis	55	26	0.024	1.374	0.122
Metastasis	17	15			
Unknown	89	14			
**Pretreatment LDH level**					
Elevated	15	12	0.062		
Normal	28	28			
Unknown	118	15.5			
**Resection**					
R0	62	19.5	< 0.001	1.045	0.678
R1	6	12.5			
R2	18	6.5			
Extent of resection unknown	50	19			
Biopsy only	25	10			
**Tumor stage**					
II	15	26	0.107		
III	33	22			
IV	43	12			
Stage unknown	70	15			
**Cycles of chemotherapy**					
> 10	32	33	< 0.001	0.370	< 0.001
4–10	77	16			
< 4	23	12			
No chemotherapy	26	13.5			
Cycles unknown	3	19			
**Dose of radiotherapy**					
> 50 Gy	24	28.5	0.376		
> 30 Gy, ≤ 50 Gy	33	18			
≤ 30 Gy	10	15.5			
No radiotherapy	94	14			
**Chemotherapy regimen**					
VDC/IE	87	18	0.017	1.082	0.814
Other regimens	42	16.5			
Regimen unknown	6	9.5			
No chemotherapy	26	13.5

**Table 2:** Comparison of data on pPNETs between previous reports (75 cases) and our institution (86 cases).

Variable	Previous reports	Our institution
**Age at diagnosis (years)**		
Median	24	23
Range	1–64	1–78
Patients aged ≤ 18y	30 (40%)	33 (38%)
Male sex	31 (41.3%)	41 (48%)
Surgery	67 (89%)	69 (80%)
Chemotherapy	70 (93%)	65 (76%)
Radiotherapy	31 (41%)	36 (42%)
Median overall survival	17 months	15.5 months

Of all patients, 84.4% underwent surgery, 83.9% had chemotherapy, and 41.6% had radiotherapy. Of those who received surgery, 46% had R0 resection, 4.4% had R1 resection, 13% had R2 resection, and the extent of surgery were not clearly documented in 37% of the surgical cases. Of those who received chemotherapy, 64.4% had VDC/IE (vincristine + doxorubicin + cyclophosphamide)/(isophosphamide + etoposide) regimen, 31.1% had regimens other than VDC/IE, and 4.5% had unknown chemotherapy regimen. Of the 132 patients whose number of cycles of chemotherapy was available, 23 received less than 4 cycles of chemotherapy, 77 received 4 to 10 cycles, 32 received more than 10 cycles. Of the 67 patients who underwent radiotherapy, 10 were given a dose of ≤ 30 Gy (two for curative purpose, one for palliation, and seven for adjuvant therapy), 33 were given > 30 Gy and ≤ 50 Gy (four for curative purpose, one for palliation, and the others for adjuvant therapy), and 24 were treated with > 50 Gy (five for curative purpose and nineteen for adjuvant therapy). Eight patients were treated with curative radiotherapy as the initial local treatment, 54 patients received postoperative adjuvant radiotherapy, four patients received curative radiotherapy after disease recurrence or progression (only one out of the four received adjuvant radiotherapy), and two were treated with palliative radiotherapy after metastatic or progressive disease. Of the 161 eligible patients, 10 patients underwent surgery only, 13 received chemotherapy only, none was treated with radiotherapy only, 4 patients had no treatment and passed away soon after diagnosis, and the others all received combination therapy.

### Survival analysis

**Figure 2:**
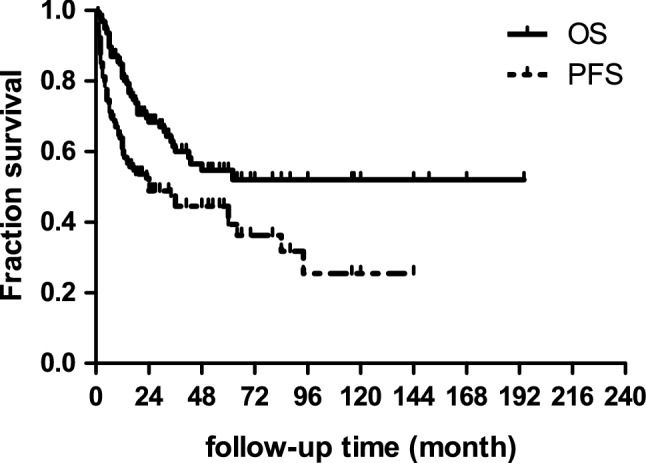
Overall survival and progression free survival of the entire cohort. The 5-year, 3-year, 1-year overall survival rates were 54%, 60%, and 81%, respectively. The 5-year, 3-year, 1-year progression free survival rates were 38%, 44%, and 59%, respectively.

The follow-up for the whole series ranged from 1 to 194 months. The mean and median overall survival (OS) of the 161 patients were 29.2 months and 16 months, and the mean and median progression free survival (PFS) were 14.4 months and 8 months. The 5-year, 3-year, 1-year overall survival rates were 54%, 60%, and 81%, respectively. Progression free survival was available in 156 cases, and the 5-year, 3-year, 1-year PFS rates were 38%, 44%, and 59%, respectively (see Fig. 2).

In the entire cohort, surgery, chemotherapy, and female sex were associated with superior OS by univariate analysis (*P* < 0.05). We assessed the impact of different modes of local control on final outcome. The 5-year OS was 56% and 61% with surgery plus radiotherapy and surgery alone, respectively. The 5-year OS was only 30% for patients without surgical resection. It is difficult to draw a definitive conclusion on the role of radical radiotherapy in the current cohort since there were only eight patients who received radiotherapy as the initial definitive treatment. Among these eight patients, two had progressive disease after chemoradiation, and two had local recurrence after initial radiotherapy. In the 54 patients who received postoperative adjuvant radiotherapy, 10 had local recurrence and 13 had progressive disease or distant metastasis. For the 49 patients who received non-R0 resection (R1/R2 resection or biopsy only), radiotherapy proved to be significantly associated with better overall survival (*P* = 0.002).

We further evaluated the significance of various clinical and treatment plans that may influence prognosis. In univariate analysis of all PNET patients, small tumor diameter, no lymph node metastasis, R0 resection, more than 10 cycles of chemotherapy, and VDC/IE regimen predicted improved OS. In multivariate analysis of the significant variables listed in Table 1, surgery, more than 10 cycles of chemotherapy, and small tumor diameter proved to be independent prognostic factors for higher OS (*P < *0.05). All the preceding factors were re-evaluated for PFS, surgery, chemotherapy, female sex and no lymph node metastasis were significant factors improving PFS (*P < *0.05). In multivariate analysis, surgery, chemotherapy, and female sex remained as independent favorable predictive factor for progression free survival.

## Discussion

Peripheral primitive neuroectodermal tumors constitute very rare and aggressive malignancies that arise in various body sites. Consequently, relevant studies are generally of small patient numbers, diverse therapeutic strategies and different survival outcomes. Generally, patients with localized disease can have a 5-year survival of 50–60%, while relapsed and metastatic patients have a 5-year survival less than 20%^9,10^. The prognosis of PNETs depends on many factors, such as patient age, tumor site, tumor volume, metastatic disease, and treatment plans. The role of patient age in predicting disease outcome remains controversial. Substantial historical data from large randomized trials and large retrospective cohorts suggests that adults with Ewing family tumors do significantly worse than children, with 5-year OS rates ranging from 20 to 60% for localized disease^11–14^. However, many of these studies have focused on patients treated in the past century, before the advent of modern VDC/IE 5 drug regimen. In recent years, some centers reported a comparable outcome of their adult patients treated with modern multimodality therapy to outcome in children^15,16^. Our study did not reveal a survival difference between age groups. One future question to answer is whether this difference in survival is caused by difference in treatment plans or intrinsic difference in tumor and/or host biology. Since past randomized clinical trials mainly focused on pediatric population, optimal therapeutic strategy for older adults diagnosed with Ewing family tumors remains to be defined.

In regards to the relationship between sex and survival, the INT-0091 trial did not report a survival difference by sex: 5-year PFS was 59% and 65% for male and female, respectively (HR 0.85, *P = *0.32)^12^. Our results showed that female patients had statistically significant better survival than male patients in univariate analysis. We speculated that the discrepancy was because patients enrolled in INT-0091 trial were all under 30 years and their malignancies were limited to Ewing sarcomas/primitive neuroectodermal tumors of bone. The heterogeneity in patient population could possibly explain the difference between study results. Survival prognosis of PNET appears to correlate with the site of the primary tumor. For example, a series of 28 patients with peripheral PNET in their limbs showed a 1-year and 2-year survival rate of 100% and 64%, respectively^17^. In contrast, purely intramedullary PNET has dismal outcome: most patients die within two years in spite of surgical excision followed by radiotherapy and chemotherapy^18^. Whether extraosseous tumor origin may be a favorable prognosis factor is still unclear^19,20^.

To date, there are no standard guidelines for management of peripheral PNETs because of the paucity of cases arising in various body sites. Therapeutic approach is derived from Ewing sarcoma family, which currently remains multimodal. For the entire series, we observed a significantly superior prognosis associated with surgery and chemotherapy. Further analysis showed that main parameters leading to higher survival included: more complete surgical resection, more than 10 cycles of chemotherapy and VDC/IE regimen. In the literature, the role of extensive and complete or near complete surgical excision has been advocated as critical for local tumor control and prolonged survival^5,21,22^, which was corroborated by our results. Retrospective analysis of several large groups gives the impression of maximal local control when surgery is feasible^23^.

Small round cell tumors such as Ewing’s sarcoma family usually respond well to radiation^24^. Therefore, radiotherapy is frequently indicated for primary and adjuvant treatment of PNET. Radiotherapy as local treatment approach is used when complete local resection is not feasible with a functional organ, a difficult anatomic location, or with very large tumor volume not amenable to radical surgery even after neoadjuvant chemotherapy, and in case of a metastatic disease. Post-operative radiotherapy has been implied to decrease local recurrence and provide prolonged survival^25,26^. The selection of local treatment modality is considerably biased by several factors, including tumor location, tumor volume, sensitivity to chemotherapy, patient general status, and institutional protocol. Until now, there have been no randomized studies comparing surgery and radiotherapy in Ewing tumors. Some studies reported that radical radiotherapy as the only local treatment for Ewing’s sarcoma predicted adverse survival or local control^24,27,28^. However, in these studies, the choice of local treatment was influenced by multiple factors and thus non-randomized. The radical radiotherapy group generally had more risk factors compared with their counterparts who underwent surgical intervention, which might explain the adverse outcome. One group controlled for known confounding factors influencing choice of local treatment and prognosis, and concluded that local treatment modality was not significantly related to PFS, OS, or distant failure, though local failure was more common for radiotherapy than surgery^28^. In another cohort of 1058 patients with nonmetastatic Ewing’s sarcoma and malignant PNET, researchers found that tumor size and site were not the only determinant of local control in irradiated patients compared with surgical patients. Even in theoretically favorable patients, definitive radiotherapy was associated with more local failures^27^. In a study of nonmetastatic chest wall Ewing sarcoma family tumors, local control was similar in patients treated with radiation alone versus patients treated with surgery with or without radiation. The authors suggested that definitive radiotherapy should be applied in every situation except when the tumor has a small primary volume and is in an operable site where a wide resection margin can be achieved^29^. A total radiation dose of 55–60 Gy is recommended for patients undergoing biopsy or R1/R2 resection^30,31^. Based on our center’s experience, when marginal or total resections are feasible, an attempt should be made to perform surgical resection. Although radiotherapy is not free from side effects at the primary tumor sites, we still suggest that radiotherapy should be conducted at least in the case of a marginal or incomplete removal.

Chemotherapy is an indispensable systemic treatment in patients with peripheral PNET. Due to the similarity between pPNET and Ewing’s sarcoma, chemotherapy regimens for pPNET are mainly extrapolated from those for Ewing’s sarcoma. At present, the recommended chemotherapy regimen includes a four-drug combination of vincristine, doxorubicin, dactinomycin, and cyclophosphamide along with additional cycles of ifosfamide and etoposide^23,32–34^. Chemotherapy protocol for Ewing sarcoma family tumors typically has two phases: neoadjuvant chemotherapy (induction) to enhance local control, and adjuvant chemotherapy (consolidation) to prevent metastasis, usually achieving a total of 14–17 cycles. Regimen should be risk-adapted, adjusting cycle number, dose and intensity according to tumor site, metastatic status, tumor resectability, surgical margin, response to induction and so on. Intensively timed vincristine–doxorubicin–cyclophosphamide (VDC) alternating with isophosphamide–etoposide (IE) is the North American standard chemotherapy regimen for patients with localized Ewing sarcoma family tumors, whereas a less intensive vincristine–ifosfamide–doxorubicin–etoposide (VIDE) induction chemotherapy followed by vincristine–dactinomycin–ifosfamide (VAI) or vincristine–dactinomycin–cyclophosphamide (VAC) consolidation is now considered the standard chemotherapy in Europe^34^. Research on the intensification of chemotherapy regimen suggested that shortening the interval between VDC/IE administrations from 3 to 2 weeks was more effective but with no increase in toxicity^35,36^. Attempts have also been made to investigate the benefit of high-dose chemotherapy compared to the standard regimen, especially in high-risk and primary disseminated cases. High dose chemotherapy generally involves adding extra therapeutic agents to the standard regimen or replacing part of it, and in many situations hematopoietic cell support is needed. Such escalating agents include melphalan, carboplatin, busulfan, carmustine, thiotepa, procarbazine, treosulfan, irinotecan, gemcitabine and docetaxel. Results regarding busulfan and melphalan (BuMel) were conflicting and indefinite, but showed a potential benefit in selected high-risk patients^32,37,38^. The recently published Euro-E.W.I.N.G.99 and Ewing-2008 results confirmed that BuMel may be an important addition to the standard care in predefined high-risk patients^39^. However, severe acute toxicities were more frequent in the BuMel group, and the incidence of secondary malignancies in long-term survivors was not reported. Studies on other agents are more limited, thus no firm conclusion could be drawn at this stage^40–43^. To date, there is no standard treatment for refractory disease. Several combinations of agents have shown promising results in retrospective or phase II studies. Topotecan plus cyclophosphamide and temozolomide plus irinotecan are most commonly used. Gemcitabine plus docetaxel and high-dose ifosfamide have also been used in this context, with variable results^23^. Our data supported the role of chemotherapy as a beneficial component in the multimodal treatment of peripheral PNET. As observed in our study, VDC/IE regimen was superior to other regimens in terms of survival.

Besides treatment modalities, clinical biochemical and immunohistological variables are also indicative of PNET prognosis. A case series by Liang et al.^44^ demonstrated that serum lactate dehydrogenase (LDH) level > 240 U/L at initial diagnosis was related to shorter survival than a level of 80–240 U/L. In the present study, 15 cases showed increased pretreatment serum LDH, others had normal or unknown values. Seven of the fifteen patients with increased LDH had lymph node or distant metastasis at diagnosis, and the elevated serum LDH level might be an accompanying feature of their tumor volume and stage. We suggest that physicians should be cautious of the possibility of distant metastasis given a markedly elevated LDH level.

Immunohistochemistry test is a widely used laboratory technique in assisting the diagnosis of primitive neuroectodermal tumors. The most specific and traditionally used biomarker for PNET is CD99, which is a cell surface glycoprotein p30/32 encoded by the MIC2 gene. It has been reported that immunopositivity rate for CD99 is over 95% in peripheral PNETs^45^. Although PNET was originally diagnosed by microscopy and immunohistochemistry, in the past one to two decades more and more importance has been placed on a genetic confirmation of this type of tumors. The classic molecular cytogenetics change of Ewing sarcoma/primitive neuroectodermal tumor is t (11;22) (q24; q12) translocation, which generates an abnormal fusion gene EWS-FLI1 with oncogenic properties. Detection of this translocation by cytogenetic and/or molecular genetic methods is unique for PNET and is increasingly considered as the “gold standard” for diagnosis^46^. Currently, there has been some progress in using lysine specific demethylase 1 (LSD1) inhibitors as a pharmacological blockage of EWS-FLI function, and this could be an attractive therapeutic strategy for Ewing sarcoma/primitive neuroectodermal tumor^47^.

The current study is a large single-institution study on peripheral primitive neuroectodermal tumors. However, it is limited by its retrospective nature, the missing of certain clinical and survival information, and we were not able to collect toxicity data in our retrospective study. For the incorporated case reports from literature search, there is institutional heterogeneity in clinical decision making. Further larger studies are warranted to confirm our findings in these patients.

## Conclusions

In summary, peripheral primitive neuroectodermal tumor is clinically aggressive and has gloomy prognosis. Because of the rarity of pPNET, a randomized prospective study of therapy for this tumor is not feasible. Nevertheless, physicians need to choose the most appropriate local and systemic therapy for each individual patient. Therefore, nonrandomized studies have to be reviewed. Multimodal therapy is the mainstay therapeutic approach for peripheral PNET. Operable cases should receive maximal safe surgical excision combined with adjuvant chemoradiation. Tolerable patients should be given reasonably intensive chemotherapy and radiotherapy in order to improve their final outcome.

## Methods

We retrospectively searched the Peking Union Medical College Hospital electronic medical record system from February 1, 1998 to February 1, 2018 and found 86 inpatient cases of peripheral primitive neuroectodermal tumors after obtaining approval from the institutional review board. Primitive neuroectodermal tumors in the central nervous system were not included in this study. The clinical data of these patients were retrospectively collected. All enrolled patients needed to have an established pathological diagnosis of primitive neuroectodermal tumor by surgical or biopsy specimens. Patients who died of non-cancer related death, lost to follow-up, or without recorded clinical date were excluded from this study. Medical records at PUMCH were retrospectively reviewed for demographic data, clinical and histo-pathologic information, and treatment parameters. Dates of death, cancer recurrence and metastasis were confirmed by either querying the medical records, or making telephone interviews. The last time of follow-up of this study was June 1, 2018. Overall survival was defined as the interval from the date of treatment initiation to the date of death by pPNET or the final follow-up, with patients alive at last follow up censored on that date. Progression free survival was defined as the interval from the date of the initial treatment to the date of first relapse or metastasis by pPNET or the final follow-up, with patients censored on the date of last follow up if alive without disease progression on that date.

The remaining patients were extracted from case reports and case-series studies in the literature. From January 2010 to June 2020, PubMed was searched using “primitive neuroectodermal tumor/tumour/cancer/carcinoma” as key words in the title or abstract. 1142 articles were identified from PubMed searching. These articles were then screened for those which provided individual peripheral PNET patient data on clinicopathologic, treatment, and survival information. Articles not on pPNET or not published in the English language were excluded. Accessible full-text articles were then assessed for eligibility. We only recruited cases which received multimodal therapy. Cases with unclear documentation of radiotherapy dose or cycle of chemotherapy were excluded. A follow-up time of at least 6 months was required. We made every effort to make sure that every included case had a clear diagnosis of peripheral PNET. When the same author’s data obtained from the same or overlapping patients in more than one publication, only the most recent report or the most complete one was selected in the analysis. Finally, 75 individual cases extracted from 53 articles were included in the current analysis.

The staging criteria of pPNET is based on the staging of soft tissue sarcoma according to the 8th edition of the American Joint Committee on Cancer (AJCC) cancer staging manual. PNET is histologically a Grade 3 tumor and it is at least stage II.

Survival analysis was conducted using Kaplan–Meier methods and log-rank tests. Multivariate analysis was carried out using Cox proportional hazards regression methods. *P* values < 0.05 were considered statistically significant. All analyses were performed using SPSS 24.0.

### Ethical approval

The Institutional Review Board (IRB) of Peking Union Medical College Hospital reviewed the protocol. All methods were performed in accordance with the relevant guidelines and regulations and the IRB approved the protocol.

### Informed consent

This is a retrospective study and the IRB waived the need for written informed consent.

## Supplementary information

Supplementary Tables.

## Data Availability

The datasets used and analyzed during the current study are available from the corresponding author on reasonable request.
