# Supplementary material for: Outcome of multidisciplinary treatment of peripheral primitive neuroectodermal tumor
Source: Sci Rep. 2020 Sep 24;10:15656. doi: 10.1038/s41598-020-72680-6 (PMC7519088; doi:10.1038/s41598-020-72680-6)
Supplement: Supplementary file 1 — Supplementary Tables. [file 41598_2020_72680_MOESM1_ESM.pdf]

Outcome of multidisciplinary treatment of peripheral primitive neuroectodermal tumor

Authors: Yidi Liu, Yan Yuan, Fuquan Zhang, Ke Hu, Jie Qiu, Xiaorong Hou, Junfang Yan, Xin Lian, Shuai Sun, Zhikai Liu, Jie Shen

Supplementary Table S1 Summary of previous cases included in the current analysis

	First author	Age	Gender	Tumor site	Follow-up (months)	Status	Treatment
1	Pratik Gurung	19	M	Spermatic cord	13	NED	S+C
2	Athanasios Krassas	48	M	Chest wall	23	NED	S+C+R
3	Anubhav Vindal	26	F	Breast	36	NED	S+C
4	Yun-jian Wu	26	F	Kidney	17	AWD	S+C
5	Mitul Parikh	5	M	Chest wall	8	NED	S+C
6	Zhengcheng Liu	13	M	Chest wall	12	NED	S+C
7	Ho Yong Choi	31	M	Cerebellopontine angle	13	DOD	S+C+R
8	Farah Farzaneh	45	F	Cervix	48	NED	S+C
9	Mahdi Aghili	3	M	Kidney	56	NED	S+C+R
10	Samrat Dutta	36	F	Paravaginal	8	NED	S+C+R
11	Suebwong Chuthapisith	46	F	Breast	7	DOD	C+R
12	N Arora	23	F	Cervix	48	NED	S+C+R
13	Aharon Gefen	16	F	Kidney	36	NED	S+C+R
14	Chuanyu Sun	45	M	Kidney	15	NED	S+C
15	Zineb Benbrahim	27	F	Chest wall	36	NED	S+C
16	Magdalena Chirila	48	M	Thyroid	1	DOD	S+C
17	Ashis Patnaik	9	M	Spinal cord	7	DOD	S+C+R
18	Tomoshige Akino	16	M	Penis	84	NED	S+C

19	Deep Dutta	40	F	Adrenal gland	8	NED	S+C
20	A Mitch Dizon	50	F	Uterus	16	NED	S+C
21	Divya Khosla	28	F	Cervix	33	NED	S+C+R
22	Marion Cole	51	F	Dura	15	NED	S+C
23	Alessandro Crestani	50	M	Seminal vesicle	120	NED	S+C+R
24	Libo Peng	36	F	Mesentery and ileocecum	34	DOD	S+C
25	Jin Yong Jeong	14	F	Cheat wall	36	NED	S+C
26	Nirmalya Chakrabarti	24	F	Kidney	15	DOD	S+C
27	Cheng Yang	31	F	Kidney	14	NED	S+C
28	Uttam K Mete	20	F	Kidney	6	DOD	S+C
29	Hiroki Yoshihara	14	F	Kidney	70	NED	S+C
30	Yang Zhang	28	F	Spinal cord	13	NED	S+C+R
31	Xin Jin	37	F	Lung	25	NED	S+C+R
32	Ivan Zokalj	32	F	Kidney	116	NED	S+C
33	C R Elizalde	60	F	Uterus	7	DOD	S+C
34	C Liu	24	F	Kidney	60	NED	S+C
35	Qing Li	17	M	Lung	30	AWD	C+R
36	Rosalba De Nola	39	F	Uterus	24	NED	S+C
37	Kourosh Goudarzipour	2	F	Adrenal gland	8	NED	S+C
38	Munetoshi Akazawa	41	F	Uterus	96	AWD	S+C+R
39	Betül Yakıştıran	64	F	Ovary	7	NED	S+C
40	Ayako Takigami	45	M	Lung	10	DOD	S+C
41	Fatemeh Nili	23	F	Ovary	12	NED	S+C
42	Tian-Qi Du	39	M	Prostate	17	DOD	C+R
43	Vipul D Yagnik	42	M	Small bowel	9	NED	S+C
44	Xiao Hu	39	F	Cervix	60	NED	S+C

45	Yachao Liu	40	M	Prostate	14	NED	S+C
46	Kusay Ayoub	20	M	Pelvic	24	NED	S+C+R
47	Rehan Mohsin	26	M	Kidney	5	DOD	S+C
48	Rehan Mohsin	34	M	Kidney	1	DOD	S
49	Hong-cheng Song	5	M	Penis	9	NED	C+R
50	Tadeusz Kroczak	17	M	Kidney	12	NED	S+C
51	Tadeusz Kroczak	31	M	Kidney	12	DOD	S+C
52	Geetha Narayanan	27	F	Kidney	15	NED	S+C
53	Geetha Narayanan	34	F	Kidney	15	DOD	S+C+R
54	Geetha Narayanan	17	F	Kidney	18	AWD	C+R
55	Geetha Narayanan	32	M	Kidney	14	AWD	S+C
56	Xiaofeng Wang	48	F	Cervix	27	NED	S+C+R
57	Xiaofeng Wang	43	F	Cervix	12	NED	S+C+R
58	Rithika Rajendran	12	M	Rib	36	NED	S+C
59	Rithika Rajendran	7	M	Humerus	5	DOD	C
60	Rithika Rajendran	4	F	Vertebrae	48	NED	S+C
61	Rithika Rajendran	6	F	Humerus	66	NED	S+C
62	Rithika Rajendran	11	M	Femur	6	DOD	S+C+R
63	Rithika Rajendran	17	M	Hip	18	NED	C+R
64	Rithika Rajendran	6	F	Rib	15	NED	S+C+R
65	Rithika Rajendran	4	M	Rib	18	NED	S+C+R
66	Rithika Rajendran	1	F	Suprascapular region	12	NED	S+C
67	Amit Kumar Singh	7	F	Orbit	35	DOD	S+C
68	Amit Kumar Singh	13	F	Orbit	48	DOD	S+R
69	Amit Kumar Singh	11	M	Dura	31	DOD	S+C+R
70	Amit Kumar Singh	9	M	Orbit	19	DOD	S+R

71	Amit Kumar Singh	21	M	Orbit	24	DOD	S+R
72	Amit Kumar Singh	18	M	Frontoparietal convexity	12	DOD	S+R
73	Amit Kumar Singh	12	F	Skull	19	DOD	S+R
74	Qiong-Qian Xu	5	F	Abdominal wall	66	NED	S+C
75	Qiong-Qian Xu	3	F	Vulva	6	DOD	S+C

AWD – Alive With Disease; C – Chemotherapy; DOD – Dead of Disease; F – Female; M – Male; NED – No Evidence of Disease; R – Radiotherapy; S-Surgery

Table S2 Summary of literature search result (on next page)

**Author**	**Year**	**Article title**
Pratik Gurung et al	2010	Primitive Neuroectodermal Tumor of the Spermatic Cord
Athanasios Krassas et al	2010	Primitive Neuroectodermal Tumor of the Thoracic Wall in a 48-year-old Man
Anubhav Vindal et al	2010	Primary Primitive Neuroectodermal Tumor of the Breast
Yun-jian Wu et al	2010	Primitive Neuroectodermal Tumor of the Kidney With Inferior Vena Cava Tumor Thrombus During Pregnancy Response to Sorafenib
Mitul Parikh et al	2011	Peripheral Primitive Neuroectodermal Tumor of the Chest Wall in Childhood: Clinico-Pathological Significance, Management and Literature Review
Zhengcheng Liu et al	2011	Primitive Chest Wall Neuroectodermal Tumor in a Pediatric Patient
Ho Yong Choi et al	2011	Ewing's Sarcoma/Peripheral Primitive Neuroectodermal Tumor in the Cerebellopontine Angle : Diagnosis and Treatment
Farah Farzaneh et al	2011	Primitive Neuroectodermal Tumor of the Cervix: A Case Report
Rehan Mohsin et al	2011	Primitive Neuroectodermal tumor/Ewing's Sarcoma in Adult Uro-Oncology: A Case Series From a Developing Country
Mahdi Aghili et al	2012	Renal Primitive Neuroectodermal Tumor: Does Age at Diagnosis Impact Outcomes?
Samrat Dutta et al	2011	Paravaginal Peripheral Primitive Neuroectodermal Tumor: A Rare Tumor
Suebwong Chuthapisith et al	2012	Ewing's Sarcoma and Primitive Neuroectodermal Tumour (ES/PNET) Presenting as a Breast Mass
N Arora et al	2012	Primitive Neuroectodermal Tumour of Uterine Cervix - A Diagnostic and Therapeutic Dilemma
Aharon Gefen et al	2012	Primitive Neuroectodermal Tumor of the Kidney With Renal Failure
Chuanyu Sun et al	2012	Primitive Neuroectodermal Tumor of the Kidney: Case Report and Review of Literature
Hong-cheng Song et al	2012	Primary Ewing's sarcoma/primitive Neuroectodermal Tumor of the Urogenital Tract in Children
Zineb Benbrahim et al	2013	Askin's Tumor: A Case Report and Literature Review
Magdalena Chirila et al	2013	Extraosseous Ewing Sarcoma and Peripheral Primitive Neuroectodermal Tumor of the Thyroid Gland: Case Report and Review
Ashis Patnaik et al	2013	Rare Case of Primary Primitive Neuroectodermal Tumour of Sacral Region in a Child and Its Follow-Up
Tomoshige Akino et al	2014	Successful Penile Reconstruction After Multimodal Therapy in Patients With Primitive Neuroectodermal Tumor Originating From the Penis
Deep Dutta et al	2013	Primitive Neuroectodermal Tumor of Adrenal: Clinical Presentation and Outcomes
A Mitch Dizon et al	2014	High Grade Primitive Neuroectodermal Tumor of the Uterus: A Case Report
Divya Khosla et al	2014	Primitive Neuroectodermal Tumor of the Uterine Cervix Diagnosed During Pregnancy: A Rare Case With Review of Literature

Marion Cole et al	2014	Peripheral Primitive Neuroectodermal Tumor of the Dura in a 51-year-old Woman Following Intensive Treatment for Breast Cancer
Alessandro Crestani et al	2014	Peripheral Primitive Neuroectodermal Tumor of Seminal Vesicles: Is There a Role for Relatively Aggressive Treatment Modalities?
Tadeusz Kroczak et al	2014	Renal Primitive Neuroectodermal Tumour: Case Series and Brief Review
Libo Peng et al	2015	Primary Primitive Neuroectodermal Tumor Arising in the Mesentery and Ileocecum: A Report of Three Cases and Review of the Literature
Jin Yong Jeong et al	2015	A Small Askin's Tumor Presenting With Early Onset of Chest Pain
Nirmalya Chakrabarti et al	2015	Primary Ewing's Sarcoma/Primitive Neuroectodermal Tumor of of Kidney - A Diagnostic Dilemma
Cheng Yang et al	2015	Renal Primitive Neuroectodermal Tumor: A Case Report
Uttam K Mete et al	2015	Fulminant Buddchiari Syndrome Caused by Renal Primitive Neuroectodermal Tumor With Inferior Vena Cava Thrombus Extending to Atrium
Hiroki Yoshihara et al	2016	Ewing sarcoma/primitive Neuroectodermal Tumor of the Kidney Treated With Chemotherapy Including Ifosfamide
Yang Zhang et al	2016	Synthetic Treatment of Intracranial Peripheral Primitive Neuroectodermal Tumor With Multiple Metastasis: A Case Report
Xin Jin et al	2016	Primitive Neuroectodermal Tumor Originating From the Lung: A Case Report
Ivan Zokalj et al	2016	Primary Renal Primitive Neuroectodermal Tumor/Ewing's Sarcoma Imaging and Pathologic Findings of a Patient With a Nine Year, Eight Month Disease Free Period: Case Report and Review of Literature
C R Elizalde et al	2016	Primitive Neuroectodermal Tumor of the Uterus
C Liu et al	2017	Renal Ewing's sarcoma/primitive Neuroectodermal Tumor: A Case Report and Literature Review
Qing Li et al	2017	Antiangiogenic Therapy for Primitive Neuroectodermal Tumor With Thalidomide: A Case Report and Review of Literature
Geetha Narayanan et al	2017	Primitive Neuroectodermal Tumors of the Kidney
Xiaofeng Wang et al	2017	Primary Primitive Neuroectodermal Tumor of the Cervix: A Report of Two Cases and Review of the Literature
Rithika Rajendran et al	2017	Paediatric Peripheral Primitive Neuroectodermal Tumour - A Clinico-Pathological Study From Southern India
Rosalba De Nola et al	2018	Clinical Management of a Unique Case of PNET of the Uterus During Pregnancy, and Review of the Literature
Kourosh Goudarzipour et al	2018	Ewing Sarcoma/Peripheral Primitive Neuroectodermal Tumor in the Adrenal Gland of a Child
Akazawa M et al	2018	Adjuvant chemotherapy for a primitive neuroectodermal tumor of the uterine corpus: A case report and literature review.
Betül Yakıştıran et al	2018	Primitive Neuroectodermal Tumor of Genital Tract in Hysterectomized Patient: A Case Report
Ayako Takigami et al	2019	Pazopanib Confers a Progression-free Survival in a Patient With Ewing's Sarcoma/Primitive Neuroectodermal Tumor of the Lung
Fatemeh Nili et al	2018	Peripheral Primitive Neuroectodermal Tumor of the Ovary: The Report of Two Rare Cases

Amit Kumar Singh et al	2018	Extraosseous Primary Intracranial Ewing Sarcoma/peripheral Primitive Neuroectodermal Tumor: Series of Seven Cases and Review of Literature
Tian-Qi Du et al	2019	Primitive Neuroectodermal Tumor of the Prostate With Notalgia and Paraplegia as the Initial Symptoms: A Case Report and Literature Review
Vipul D Yagnik et al	2019	Extraskeletal Ewing's sarcoma/peripheral Primitive Neuroectodermal Tumor of the Small Bowel Presenting With Gastrointestinal Perforation
Xiao Hu et al	2017	Pathologic Characteristics of Primary Primitive Neuroectodermal Tumor in the Cervix: A Small Round Cell Malignant Tumor With Glial and Epithelial Differentiation After Neoadjuvant Chemotherapy
Yachao Liu et al	2020	Primary Peripheral Primitive Neuroectodermal Tumor of the Prostate on 18F-DCFPyL PET/CT
Kusay Ayoub et al	2020	A Rare Case of Pelvic Primitive Neuroectodermal Tumor With Misleading Symptoms: A Case Report
